# Evaluation of Autonomic Nervous System, Saliva Cortisol Levels, and Cognitive Function in Major Depressive Disorder Patients

**DOI:** 10.1155/2018/7343592

**Published:** 2018-04-02

**Authors:** Sukonthar Ngampramuan, Puttichai Tungtong, Sujira Mukda, Apichat Jariyavilas, Chanin Sakulisariyaporn

**Affiliations:** ^1^Research Center for Neuroscience, Institute of Molecular Biosciences, Mahidol University, Nakornpathom, Thailand; ^2^Mahidol International College, Mahidol University, Nakornpathom, Thailand; ^3^Srithanya Hospital, Department of Mental Health, Ministry of Public Health, Nonthaburi, Thailand; ^4^Department of Psychiatry, Panyananthaphikkhu Chonprathan Medical Center, Srinakharinwirot University, Nonthaburi, Thailand

## Abstract

Major depressive disorder (MDD) is associated with changes in autonomic nervous system (ANS) and cognitive impairment. Heart rate variability (HRV) and Pulse pressure (PP) parameters reflect influences of the sympathetic and parasympathetic nervous system. Cortisol exerts its greatest effect on the hippocampus, a brain area closely related to cognitive function. This study aims to examine the effect of HRV, PPG, salivary cortisol levels, and cognitive function in MDD patients by using noninvasive techniques. We have recruited MDD patients, diagnosed based on DSM-V-TR criteria compared with healthy control subjects. Their HRV and PP were measured by electrocardiogram (ECG) and photoplethysmography (PPG). Salivary cortisol levels were collected and measured on the same day. MDD patients exhibited elevated values of mean HR, standard deviation of HR (SDHR), low frequency (LF) power, low frequency/high frequency (LF/HF) ratio, mean PP, standard deviation of pulse pressure (SDPP), and salivary cortisol levels. Simultaneously, they displayed lower values of mean of R-R intervals (mean NN), standard deviation of R-R intervals (SDNN), high frequency (HF) power, and WCST scores. Results have shown that the ANS of MDD patients were dominated by the sympathetic activity and that they have cognitive deficits especially in the domain of executive functioning.

## 1. Introduction

Historically, mental illness was viewed as a purely psychological phenomenon. However, advancement in neurology has changed this view by revealing that many physiological manifestations result from the development of mental illness. One significant physiological marker is autonomic dysregulation, which is commonly found in population with clinical depression. Several studies have shown that clinical depression has frequently been associated with autonomic dysregulation with an overall decrease in total heart rate variability, a low high frequency heart rate variability, and vagal control as distinct feature [1]. In addition, clinical depression has been classified as a strong risk factor for cardiac morbidity and mortality in coronary heart disease patients [2], as well as postmyocardial infarction patients [3]. Since psychological factors impose such a great impact on both autonomic regulation and the physical condition of the heart, the analysis of heart rate variability using electrocardiography and pulse pressure by fingertip photoplethysmography—which is the direct representation of cardiac chronoscopic control—will greatly clarify the link between major depressive disorder and autonomic dysregulation. Another significant physiological manifestation of major depressive disorder involves the so-called stress hormone, cortisol. It has been found that patients with major depressive disorder exhibited an elevated level of cortisol [4], which might be a result of hypothalamic-pituitary-adrenal (HPA) axis dysregulation [5]. This elevated level of cortisol has been associated with both psychological and physiological impacts. The elevation of mean cortisol levels in psychotic major depressive disorder patients corresponded to greater cognitive impairment in verbal memory [6]. An elevation in mean cortisol levels can be associated not only with memory impairment but also with various indices of executive functioning [7]. In terms of physiological impact, another review study has found that there is a strong relationship between elevated cortisol levels, depression, abdominal obesity, and loss of bone density [5]. Since cortisol and the autonomic nervous system function in a coordinated manner in responding to a stressful event [8], an investigation of the relationship between cortisol and major depressive disorder will allow a better comprehensive understanding of autonomic nervous system activity in patients who suffer from this psychiatric condition.

Furthermore, depression is considered to be the most important risk for suicide—accounted for 0.9% of all deaths—with about two-thirds of suicide occurring in MDD patients [9]. Although clinical depression is a life-threatening mental epidemic, the rate of misdiagnosis is relatively high. A meta-analysis from over 50,371 patients across 41 studies in the United Kingdom has suggested that, for every 100 unselected cases of unassisted diagnosis (without the use of severity scales, diagnostic instruments, education programs, or other organizational approaches) seen in primary care, there are more false positives (*n* = 15) than either missed (*n* = 10) or identified cases (*n* = 10) [10]. The possible causes of this problem might be the time period before official diagnosis and the deficiency of an objective physiological diagnostic tool. Therefore, the analysis of heart rate variability, pulse pressure, and cortisol level in major depressive patients, which contributes to the development of objective diagnostic and prognostic prediction tools, is of great importance. The aim of this study is to examine the efficacy of the biomarkers, electrocardiogram (ECG), and photoplethysmography (PPG), in order to evaluate the measurements of the heart rate variability (HRV), pulse pressure (PP), and salivary cortisol level from noninvasive techniques as a supplementary diagnostic tools for major depressive disorder in Thailand.

## 2. Materials and Methods

### 2.1. Participant

Major depressive disorder is one of the most common chronic conditions that throughout the lifetime 17% of the adult population may have developed [11]. The age of onset distribution of mood disorders (major depression, dysthymic disorder, and bipolar disorder) is 29–43 years old, reported by the World Health Organization's (WHO) World Mental Health (WMH) [12]. The participants recruited to this research were all adults, aged between 20 and 43 years old, both male and female (to avoid effects of sexual difference), with a total of 40 participants, 20 participants in the MDD group and 20 participants in the control group. Participants in the MDD group (MDD patients) were all evaluated by psychiatrists from the Panyananthaphikkhu Chonprathan Medical Center with DSM-V-TR criteria, using diagnostic interview as an initial screening for unrelated psychiatric conditions. In contrast, participants in the control group must have had no past or current history of any psychiatric illness. All participants had no past or current history of cardiovascular disease, chronic disease (e.g., cancer, epilepsy, diabetes, or stroke), or any other chronic medical conditions that are risk factors for cardiovascular disease (e.g., hypertension, hypercholesterolemia, or obesity), or substance abuse or dependence (e.g., alcoholism, nicotine addiction, or narcotics addiction). Any participants who had undergone neurosurgical procedure or sustained a head injury before were excluded from the study. Prior to data collection, all participants were informed about the details of this study and were asked to sign their informed consent, approved by the Panyananthaphikkhu Chonprathan Medical Center (No. EC 021/58) and Mahidol University Ethics Committee (COA No. MU-CIRB 2016/103.1608).

### 2.2. Instruments

#### 2.2.1. Electrocardiogram

Electrocardiogram (ECG) was measured by using a Bluetooth-operating telemetric monitor called BIOPAC BioHarness (USA), which can log or view a variety of parameters such as ECG raw signal, breathing data, R-R interval of ECG signal, heart rate, respiratory rate, skin temperature, posture, and breathing wave amplitude with radio frequency (RF) transmission [13]. Be advised that BioHarness requires AcqKnowledge™ software and a computer with integrated Bluetooth or an external USB Bluetooth dongle to operate.

#### 2.2.2. Fingertip Photoplethysmography

Fingertip photoplethysmography (PPG) was measured using BIOPAC MP150 System and its probes. The MP150 unit will register incoming signals from the probe and convert them into digital signal that will be processed by a computer [14]. It is a complete data acquisition system, which includes all hardware and software (AcqKnowledge) required for the system to operate on any computer.

#### 2.2.3. Saliva Sample Collection

Saliva samples were collected in sterilized 1.5 mL Eppendoft® Safe-Lock microcentrifuge tubes, which are made of polypropylene. Samples were gathered at hospital on a regular working day shortly after the baseline interview was conducted. Subjects were instructed to refrain from eating, smoking, drinking tea or coffee, and brushing teeth 15 minutes prior to saliva collecting and no dental work is allowed in the 24 hours before sample collection. On the collecting day, samples were taken at site, refrigerated after the collection, and brought back to Mahidol University's laboratory.

#### 2.2.4. Wisconsin Card Sorting Test (WCST)

WCST is Cognitive function measurement; a computerized version of the licensed WCST was used in this study to check for cognitive impairment in MDD. The WCST consists of 4 stimulus cards and 128 response cards. They are different in forms (crosses, circles, triangles, or stars), colors (red, blue, yellow, or green), and number of figures (one, two, three, or four) [15]. In order to solve the test, participants have to choose one of the four types of response cards that match a stimulus card in shape, color, or number. The computer then determines whether the response is correct. When 10 consecutive correct responses are made, the sorting criteria will shift without notice, and participants have to adapt a new sorting strategy in order to get another set of correct response [15]. The computerized version of the WCST contains 11 measures including the number of categories completed, the number of trials, the number of correct responses, the number of errors, the number of perseverative responses, the number of perseverative errors, the number of nonperseverative errors, the number of trials to complete the first category, the percentage of conceptual response, failure to maintain set, and learning to learn [16]. Note that the WCST software was obtained legally without any violation of its copyrights.

### 2.3. Procedure

#### 2.3.1. Heart Rate Variability and Photoplethysmography Measurements

The heart rate (HR), SDHR, LF, HF, LF/HF ratio, mean NN, and SDNN were measured using the ECG measurement. Mean pulse pressure (mean PP) and standard deviation of pulse pressure (SDPP) were obtained through the PPG measurement. Participants were asked to wear a BIOPAC BioHarness device, which came in the form of a belt, on their bare chest, right under the end of the sternum. After participants had put on the BIOPAC BioHarness belt, research staff asked them to breathe normally in order to determine whether the belt was too tight or too loose. This was done to ensure that the belt is the most comfortable fit for the participants. Participants were allowed to put their shirt back on while the system was logging data. For PPG measurement, research staff affixed the PPG sensor to the participants' nondominant index finger and left it on until the data logging was done. The PPG operated on the reflection mode to obtain a pressure pulse wave from participants' finger pulse profile. Moreover, the experiment was conducted in a room with identical lighting condition for all participants to minimize the effect of ambient light inferences.

The ECG and PPG recording for each participant started simultaneously from the beginning of the resting period until the end of the recovery period; hence the ECG and PPG recording lasted about 55–60 minutes. The data recorded from ECG and PPG measures were processed and analyzed by the accompanying software provided with the system. Both procedures are entirely noninvasive as no gel or puncture is required.

Note that, in order to minimize the effect of motion artifact that may have resulted from the participants' movements while completing the WCST test, both ECG and PPG devices and their peripherals were place on the opposite side of the computer mouse.

#### 2.3.2. Salivary Cortisol Measurement

*Saliva Sample Preparation.* Measurement of active free cortisol was performed using the enzyme immunoassay (EIA) method. Samples were prepared by thawing to room temperature, spinning on a vortex mixer, and centrifuging at 3000 revolutions per minute (rpm) for 15 minutes in order to remove mucins and other particulate matter, which might affect the immunoassay results.

*Salivary Cortisol Enzyme Immunoassay.* In the laboratory, saliva tubes were centrifuged at 2000 grams for 10 minutes, aliquoted, and stored at −80°C. Cortisol analysis was carried out using competitive immunoassay Salimetrics®, USA. A detailed description of cortisol measurement can be referred to by the Standard Salimetrics Cortisol Enzyme Immunoassay Kit Protocol from Salimetrics company, USA [17]. Lastly, the plate was read in a plate reader at 450 nm for enzyme immunoassay results.

#### 2.3.3. Cognitive Function Test

**Figure 1:**
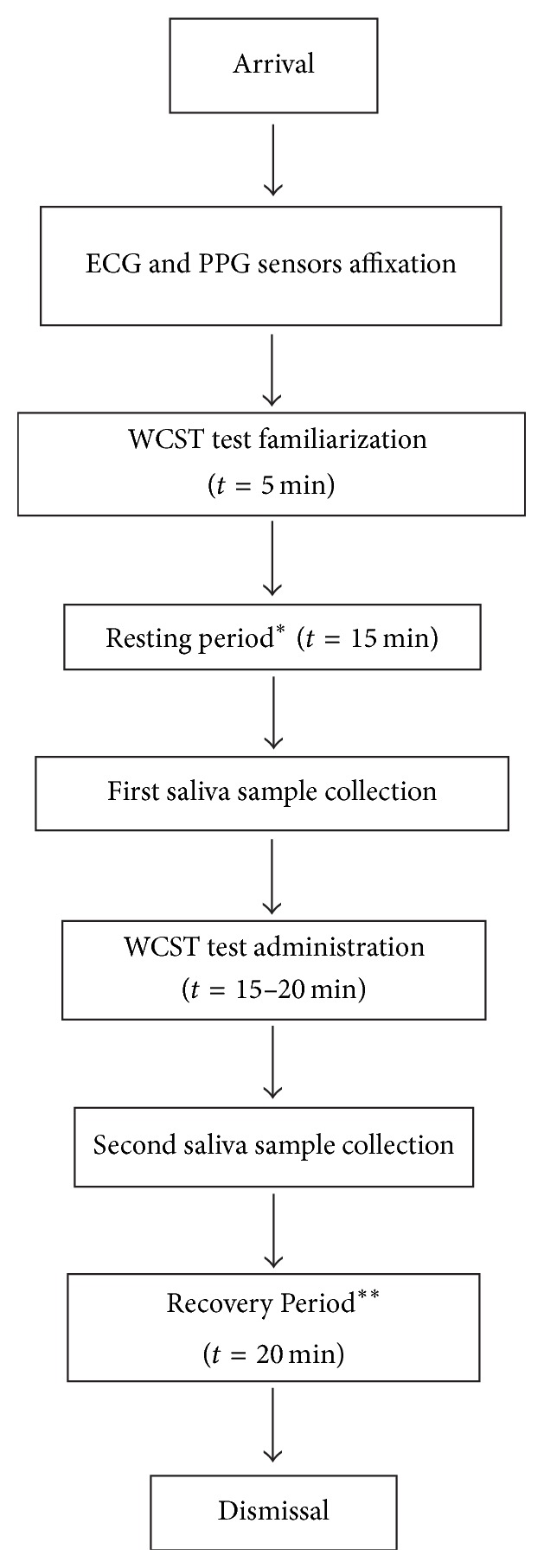
Experiment protocol. After the participants arrived, ECG and PPG sensors were placed on their chests and index finger. They were allowed to become familiar with WCST for 5 min and then started the recording of ECG and PPG during resting period for 15 min. After that saliva was collected from them for first time collection. They were allowed to conduct WCST test for 15–20 min and right after their finishing, the second saliva sample was collected. Participants were then allowed to relax on the chair for the next 20 min in the recovery period. After the recovery period, the ECG and PPG recording ended and the participants were dismissed. ^*∗*^The beginning of ECG and PPG recording. ^*∗∗*^The end of ECG and PPG recording. ECG: electrocardiogram; PPG: photoplethysmography; WCST: Wisconsin card sorting test; *t*: time; min: minutes.

Cognitive functioning was measured by the WCST. Participants with ECG and PPG sensors equipped were seated in front of a computer and were instructed as little as possible throughout the test. Note that participants used their dominant hands to complete the test and were allowed to move them freely to ensure that their WCST scores were not affected. For the first 5 minutes, participants were asked to try using the WCST test in order to become accustomed to the test, also to prevent any potential causes of a spike in the salivary cortisol which can lead to an inaccurate experimental result when the real WCST was conducted. After the participants become familiar with the WCST test, the resting period began; participants were told to relax for the next 15 minutes. At the beginning of the resting period, the ECG and PPG recording started. Right after the resting period, the first saliva sample was immediately collected. Then, the WCST was administered, 15–20 minutes into logging time. The second saliva sample was collected immediately after the test was over. Participants were then allowed to relax on the chair for the next 20 minutes, in the recovery period. At the end of the recovery period, the ECG and PPG recording was terminated and the participants were dismissed (see experiment protocol Figure 1).

### 2.4. Medication Used in MDD Patients.

This study also examined the prescribed medication for the MDD patients (as shown in Table 1) The table shows the medication category (Antidepressant, Benzodiazepine, Mood stabilizer, Antipsychotic, and Augmentation), with the minimum and maximum of the medication dosage. The percentage was calculated using the formula below:

(1) Percentage=Total participants on each medicationN=20×100.

*Remark.* A Patient might take more than one medication of the same categories.

### 2.5. Statistical Analysis

Using the GraphPad Prism to calculate and compare the significant difference between the control group and MDD group's mean age with Mann–Whitney test and Paired *t* test for HR, HRV, PPG, salivary cortisol levels, and the WCST score. The results are expressed as mean ± SEM of the individual values from each test. Statistical significance is set at *p* < 0.05.

## 3. Results

### 3.1. Demographical Data of the Participants

The mean age of the participants in the control group was 29.00 ± 1.32 years, while in the MDD group the mean age was 33.95 ± 1.58 years. There were 7 males and 13 females in the both control group and MDD group. These demographics are shown in Table 2.

### 3.2. Heart Rate Variability (HRV)

HRV data acquisition and analysis were carried out using a software named BioHarness Physiological Monitoring System, from BIOPAC Systems, Inc. Each data file was filtered to remove low frequency composite oscillating waves. Since the QRS complex waves—the ECG signals that represent the depolarization of the ventricles—of the HRV were high frequency waves, the applied filter highlighted the QRS peaks in the electrocardiogram and also facilitated selection in later parts of the program. In addition, the program recognized peaks of each graph, and other outliers caused by irregularities were eliminated through the use of a threshold. Results of all HRV parameters including mean HR, SDHR, low frequency component (LF), high frequency component (HF), LF/HF ratio, mean NN, and SDNN are shown in Table 3.

### 3.3. Time Domain Analysis

The time domain analysis of HRV took mean HR, SDHR, Mean NN, and SDNN into account. It was found that the mean heart rate of participants in the MDD group was significantly higher than the participants in the control group (MMD: 87.42 ± 3.17 BPM, control: 73.48 ± 2.01 BPM,* p* value = 0.0045). The elevation of mean HR in MDD group was also accompanied by a significantly higher SDHR (MDD: 11.67 ± 1.65, control: 5.49 ± 0.46,* p* value = 0.0017). Patients with MDD showed a significantly lower value of mean NN when compared with normal participants (MDD: 0.73 ± 0.05 ms, control: 0.87 ± 0.02 ms,* p* value = 0.0286). For SDNN, patients with MDD exhibited a significantly lower value of 56.52 ± 1.04 ms, whereas normal participants displayed a value of 68.66 ± 0.52 ms (*p* < 0.0001) as shown in Table 3.

### 3.4. Frequency Domain Analysis

Spectral analysis of HRV was characterized into three frequency domains as follows:

1. High frequency (HF) component (0.15 Hz–0.40 Hz): predominantly vagal
2. Low frequency (LF) component (0.04 Hz–0.15 Hz): predominantly sympathetic
3. LF/HF ratio: sympathovagal balance

For the high frequency (HF) component, MDD patients displayed a significant decrease in HF value when compared with normal subjects (MDD: 0.40 ± 0.03 Hz, control: 0.63 ± 0.02 Hz, *p* < 0.0001). The low frequency (LF) component value of participants in the MDD was significantly higher than participants in the control group (MDD: 0.60 ± 0.03 Hz, control: 0.36 ± 0.02, *p* < 0.0001). MDD patients consequently exhibited a significantly larger LF/HF ratio than the normal subjects (MDD: 1.77 ± 0.17, control: 0.64 ± 0.08, *p* < 0.0001) as shown in Table 3.

### 3.5. Photoplethysmography (PPG)

The results from fingertip PPG showed that patients with MDD had a significantly higher value of mean PP—a measurement of continuous blood pressure—than normal subjects (MDD: 48.92 ± 0.78, control: 33.31 ± 1.40, *p* < 0.0001). Similar results were found for SDPP, participants in the MDD group exhibited an elevated SDPP value of 8.31 ± 0.88 mmHg, while it was only 2.40 ± 0.41 mmHg for participants in the control group (*p* < 0.0001) as shown in Table 4.

### 3.6. Salivary Cortisol Levels and Wisconsin Card Sorting Test Scores

**Figure 2:**
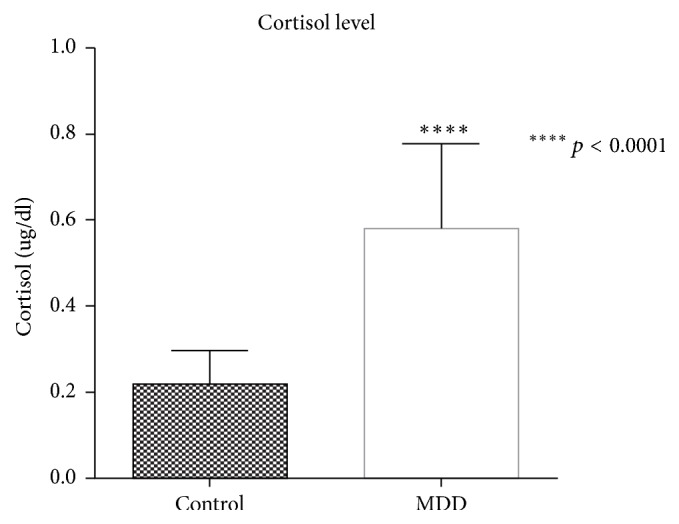
Comparison of salivary cortisol levels between control group and MDD group. Salivary cortisol level from patients with MDD showed a significantly higher level when compared with normal subjects (*p* < 0.0001).

**Figure 3:**
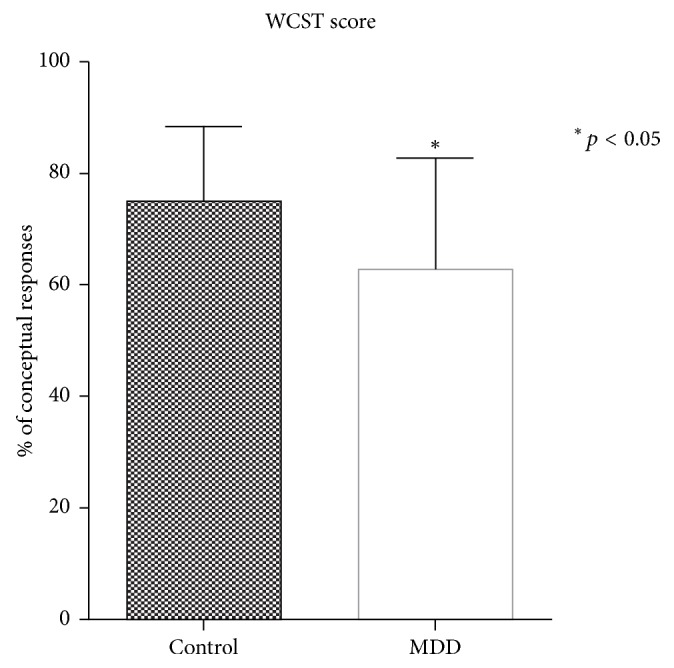
Comparison between WCST scores of control group and MDD group. WCST showed a significantly higher level of % of conceptual responses in normal subjects when compared with MDD group (*p* < 0.05). WCST: Wisconsin card sorting test.

The neuroendocrine results were obtained from salivary cortisol immunoassay, and the cognitive impairment results were acquired from the WCST. For salivary cortisol level, it was found that saliva samples from patients with MDD contained a significantly higher level of cortisol hormone when compared with normal subjects (MDD: 0.5803 ± 0.045 *μ*g/dl, control 0.22 ± 0.017 *μ*g/dl, *p* < 0.0001) as shown in Figure 2. For WCST score, participants in the MDD group performed significantly poorer than participants in the control group (MDD: 62.69 ± 4.48% of conceptual response, control: 74.6 ± 3.11% of conceptual response, *p* value = 0.0132) as shown in Figure 3.

## 4. Discussion

In this study, we aimed to examine three things: (1) the difference in HRV and PPG among healthy population and MDD patients, (2) the relationship between cortisol level and cognitive impairment in MDD patients, and (3) the efficacy of electrocardiogram, photoplethysmography, and salivary cortisol measurement as supplementary diagnostic tools for major depressive disorder.

Regarding the difference in HRV between the two groups, the time domain analysis of the acquired HRV data revealed that MDD patients have remarkably higher mean HR and SDHR values, and notably lower mean NN and SDNN values when compared to the normal subjects. These findings are similar to the review study by Berntson and Cacioppo [1]; an overall reduction of HRV (i.e., mean NN and SDNN) suggests that the cardiac chronotropic control in MDD patients is less flexible than that of normal subjects. The frequency domain analysis of HRV—which is directly associated with ANS activity—showed that MDD patients had a significant decrease in HF values (i.e., parasympathetic nervous system (PNS) modulation), an increase in LF values (i.e., sympathetic nervous system (SNS) modulation), and a larger LF/HF ratio (i.e., sympathovagal balance) when compared with normal subjects. The increase in LF values indicates that the ANS activity of MDD patients is predominantly controlled by the SNS. Since the branches of ANS function in a reciprocal manner, hyperactivation of SNS will reduce the activity of PNS; thus the HF values are lowered. In addition, as the difference between the LF and HF values gets bigger, it affects the LF/HF ratio. An increase in LF values, a decrease in HF values, and a larger LF/HF ratio have also been observed in a study by Udupa et al. [18]. Furthermore, PPG of MDD group had significant increase in mean pulse pressure (mean PP) and standard deviation of pulse pressure (SDPP) when compared with the control group that reflect pulsatile changes in blood; it can be used to estimate vascular compliance on the pressure–volume loop [19]. The hyperactivation of SNS might also be responsible for an elevation of mean HR, SDHR, mean PP, and SDPP in MDD patients.

As for the relationship between cortisol level and cognitive impairment, the results demonstrated that cortisol levels in morning saliva of MDD patients are outstandingly higher than in normal subjects. This elevation of salivary cortisol levels was accompanied by poorer performance on the conceptual response measure of WCST. These findings match the results from a study by Egeland et al., which stated that high level of morning salivary cortisol is associated with impairment in executive functioning [7]. A possible explanation for cognitive impairment—especially in executive functioning—is the reduction of cerebral blood flow to the frontal cortex of the brain as found in recent brain imaging of MDD patients [20].

To summarize, the efficacy of ECG, PPG, and salivary cortisol measurement as supplementary diagnostic tools for MDD results indicated that MMD patients are clearly distinguished from normal subjects in both HRV and salivary cortisol profile. Henceforth, it is possible to further develop these measurements into supplementary diagnostic tools for MDD in Thailand.

## 5. Conclusion

This study has conducted an analysis of heart rate variability, photoplethysmography, and its application in clinical physiological measurement—salivary cortisol levels and cognitive impairment—in two groups of subjects, one major depressive disorder group and one control group. The results have shown that the autonomic nervous system of major depressive patients is dominated by the sympathetic activity as manifested in an overall decrease of heart rate variability, a reduction of high frequency component, an elevation of low frequency component, a larger low frequency/high frequency ratio, and an increased mean and standard deviation of pulse pressure. Major depressive disorder patients also exhibited greater salivary cortisol levels and this occurrence coincided with the deficits in executive functioning as assessed by the Wisconsin Card Sorting Test. Furthermore, since the heart rate variability pulse pressure and salivary cortisol profile of major depressive disorder patients are significantly distinct from normal subjects, these measures can be further developed into supplementary diagnostic tools for major depressive disorder.

## Figures and Tables

**Table 1:** The maximum and minimum dose of medication used and proportion of patients on each medications (*n* = 20).

Medication category	Total participants on medication (*n* = 20)	%	Minimum dose (mg)	Maximum dose (mg)
*Antidepressant*				
Amitriptyline	3	15	10	25
Fluoxetine	4	20	20	20
Imipramine	1	5	25	25
Nortriptyline	3	15	25	25
Sertraline	12	60	50	50
Trazodone	3	15	50	50
*Benzodiazepine*				
Alprazolam	2	10	0.25	0.25
Clonazepam	4	20	2	2
Clorazepate	5	25	5	5
Diazepam	1	5	10	10
Lorazepam	11	55	0.5	1
*Mood stabilizer*				
Sodium valproate	3	15	200	500
*Antipsychotic*				
Risperidone	1	5	2	2
Clozapine	1	5	100	100
*Augmentation*				
Methylphenidate	1	5	10	10

**Table 2:** Demographics of the participants.

	Control group	MDD group
Number of participants (*n*)	20	20
Male (*n*)	7	7
Female (*n*)	13	13
Mean age (years)	29.00 ± 1.32	33.95 ± 1.58
Mean ± SEM		

MDD: major depressive disorder.

**Table 3:** Results of heart rate variability (HRV) in time domain and frequency domain analysis from ECG recording.

Domains	Control group	MDD group	*p* value
	Mean ± SEM	Mean ± SEM	
Mean HR (beat/min; BPM)	73.48 ± 2.01	87.42 ± 3.17^b^	0.0045
SDHR	5.49 ± 0.46	11.67 ± 1.65^b^	0.0017
Mean NN (ms)	0.87 ± 0.02	0.73 ± 0.05^a^	0.0286
SDNN (ms)	68.66 ± 0.52	56.52 ± 1.04^c^	<0.0001
HF (Hz)	0.63 ± 0.02	0.40 ± 0.03^c^	<0.0001
LF (Hz)	0.36 ± 0.02	0.60 ± 0.03^c^	<0.0001
LF/HF (Hz)	0.64 ± 0.08	1.77 ± 0.17^c^	<0.0001

*Note.* The time domain analysis of HRV showed both mean HR and SDHR in the MDD group was significantly higher than the control group whereas MDD showed a significantly lower value of mean NN and SDNN when compared with normal participants. For frequency domain analysis, MDD patients displayed a significant decrease in HF value when compared with normal subjects. Contrastingly, MDD group showed significantly higher LF component value than participants in the control group. MDD patients consequently exhibited a significantly larger LF/HF ratio than that of the normal subjects; ^a^significant difference between the control group and MDD group (*p* < 0.05); ^b^high significant difference between the control and MDD group (*p* < 0.001); ^c^high significant difference between the control and MDD group (*p* < 0.0001); HRV: heart rate variability; ECG: electrocardiogram; HR: heart rate; SDHR: standard deviation of heart rate; MDD: major depressive disorder; mean NN: mean of R-R intervals; SDNN: standard deviation of R-R intervals; HF: high frequency power; LF: low frequency power; LF/HF: low frequency/high frequency.

**Table 4:** Results of photoplethysmography.

Domains	Control group	MDD group	*p* value
	Mean ± SEM	Mean ± SEM	
Mean PP (mmHg)	33.31 ± 1.40	48.92 ± 0.78^c^	<0.0001
SDPP (mmHg)	2.40 ± 0.41	8.31 ± 0.88^c^	<0.0001

*Note. *Photoplethysmography showed mean PP and SDPP were significantly higher in MDD participants than control; ^c^high significant difference between the control and MDD group (*p* < 0.0001); PPG: photoplethysmography; mean PP: mean of pulse pressure; SDPP: standard deviation of pulse pressure; mmHg: millimeters of mercury.

## Additional Points

*Limitations and Further Studies*. The methodological limitation of this study was that our HR, HRV, PPG, cortisol, and WCST dataset contains a total of 20 subjects in each group, where the sample size of each parameter is too small to calculate with the sensitivity and specificity test. As we have found the significant differences among them, these parameters could be a possible biomarker of this disease in the near future. Therefore, further research should investigate the diagnosis test of sensitivity, specificity, and predictive values in all parameters in a larger cohort of MDD patients, in order to confirm the biopotential from the ANS responses, cognitive and cortisol, which will enable an appropriate evaluation. Moreover, further studies should expand the measurement in the different antidepressant usage, in correlation with the parameters, overtime of MDD patients.

## Supplementary Materials

Supplementary Materials Table 1: raw data from 20 subjects in control group. Table 2: raw data from 20 subjects in MDD group. Optional Table 3: previous raw data of 30 subjects in MDD group (before cut off participants of age above 43 years old).
