# Supplementary material for: Evaluation of Autonomic Nervous System, Saliva Cortisol Levels, and Cognitive Function in Major Depressive Disorder Patients
Source: Depress Res Treat. 2018 Apr 2;2018:7343592. doi: 10.1155/2018/7343592 (PMC5902046; doi:10.1155/2018/7343592)
Supplement: Supplementary Materials — Table 1: raw data from 20 subjects in control group. Table 2: raw data from 20 subjects in MDD group. Optional Table 3: previous raw data of 30 subjects in MDD group (before cut off participants of age above 43 years old). [file 7343592.f1.pdf]

Supplement Data

Table 1: Raw Data from 20 Subjects in Control Group.

SUBJECT CODE	AGE (years)	GENDER	HR (BPM)	SDHR	MEAN NN (ms)	SDNN (ms)	HF (Hz)	LF (Hz)	LF/HF (Hz)	MEAN PP (mmHg)	SDPP (mmHg)	CORTISOL (Ug/dL)	WCST (%ctr)
**C001**	26	F	70.6233	5.767	0.897264	69.10113	0.437009	0.462991	0.639543	31.80856	4.910868	0.329	69
**C002**	24	F	65.79066	5.3097	0.898233	66.20749	0.55049	0.40051	0.271312	33.1223	3.607598	0.271	70
**C003**	26	M	85.40858	2.355	1.142241	68.50932	0.663971	0.336029	0.902167	43.9908	4.307508	0.213	87
**C004**	20	F	63.86703	6.557902	1.097294	66.31666	0.624424	0.475576	0.318039	26.47209	2.833577	0.262	90
**C005**	37	M	66.64838	5.082936	0.837319	66.49437	0.584877	0.415123	0.54609	26.17112	2.350638	0.225	92
**C006**	32	F	72.04973	2.980324	0.945333	70.24593	0.661709	0.338292	0.749761	28.22627	2.720826	0.188	89
**C007**	25	F	65.51028	2.994	0.956729	67.19644	0.73854	0.26146	0.394023	24.84106	0.115914	0.356	64
**C008**	21	F	61.77083	6.159415	0.990903	71.00621	0.70661	0.277425	0.455207	24.91688	0.162696	0.2995	46
**C009**	22	F	73.85259	2.500113	0.871986	75.44298	0.679892	0.29339	0.510822	25.02322	0.322752	0.243	77
**C010**	20	M	61.54492	8.534771	0.823552	68.67994	0.661688	0.320108	0.551286	43.71474	3.267245	0.265	90
**C011**	32	F	73.4	6.619427	0.782548	68.32869	0.831555	0.338312	0.242567	43.9664	2.398398	0.243	67
**C012**	34	M	71.85	2.7113	0.814429	65.17105	0.683774	0.168445	1.10708	29.95773	2.865005	0.221	87
**C013**	35	M	80.42	6.115804	0.726404	70.12877	0.625178	0.516226	0.639543	36.59945	1.882384	0.101	67
**C014**	36	F	89.7	6.165872	0.959392	69.49337	0.4655	0.374822	1.18823	36.19816	1.560425	0.1795	77
**C015**	37	F	88.46	5.960649	0.757473	67.29231	0.74384	0.5345	0.384375	38.93836	1.807217	0.258	86
**C016**	25	F	77.74	3.989396	0.663734	70.39447	0.636786	0.25616	0.610385	34.42475	0.570609	0.263	83
**C017**	28	F	78.37	5.91883	0.798856	66.20343	0.404624	0.363214	1.51143	34.52584	0.701798	0.1975	80
**C018**	34	F	68.65	5.900226	0.897513	67.32389	0.520448	0.595376	0.961423	34.66762	0.208501	0.132	50
**C019**	34	M	66.53	9.069541	0.876678	68.98138	0.737465	0.262535	0.395997	33.23969	6.554491	0.096	65
**C020**	32	M	87.45	9.23229	0.680577	70.58475	0.70528	0.29472	0.457877	35.34168	4.816797	0.06	56

Table 2: Raw Data from 20 Subjects in MDD Group

SUBJECT CODE	TYPE	AGE	GENDER	HR	SDHR	MEAN NN	SDNN	HF	LF	LF/HF	MEAN PP	SDPP	CORTISOL	WCST
		(years)		(BPM)		(ms)	(ms)	(Hz)	(Hz)	(Hz)	(mmHg)	(mmHg)	(Ug/dL)	(%ctr)
**MDD001**	Moderate	34	Male	100.2094	22.082	0.600007	52.97838	0.285452	0.683723	2.61242	51.48396	11.56542	0.75	85.69
**MDD003**	Moderate	24	Female	94.00608	11.04298	0.640209	53.09763	0.365145	0.621951	1.898547	50.42157	9.885724	0.638667	84.69
**MDD004**	Moderate	34	Male	94.08978	12.03713	0.637004	53.92932	0.361839	0.642988	1.96417	50.65219	9.885724	0.64	47.69
**MDD007**	Severe	33	Female	102.9251	21.10614	0.583077	52.65059	0.27251	0.744215	2.678893	52.26078	12.37064	0.853333	82.69
**MDD008**	Moderate	41	Female	89.683	8.393	0.680418	55.09763	0.393865	0.580973	1.595653	49.73191	9.29594	0.542667	85.69
**MDD009**	Moderate	41	Male	101.4409	21.10614	0.589044	52.6884	0.283049	0.701513	2.678893	52.12959	11.56542	0.8	51.69
**MDD013**	Severe	40	Female	89.98518	9.9	0.6744	54.92196	0.389065	0.593317	1.75032	49.77614	9.797434	0.542667	98.69
**MDD014**	Moderate	22	Female	96.33837	16.40111	0.62906	53.97838	0.355791	0.67314	1.96417	50.96069	10.12024	0.672	36.69
**MDD015**	Moderate	35	Female	83.96518	7.009919	0.698949	56.10535	0.445193	0.549209	1.50184	47.82162	7.555456	0.439	58.69
**MDD017**	Mild	42	Female	82.05428	6.08	0.701038	56.92932	0.47674	0.541071	1.37891	47.64733	5.818441	0.41	72.69
**MDD018**	Moderate	43	Female	64.95774	3.008218	1.336461	63.12753	0.565869	0.458841	0.49013	42.94451	1.903315	0.343	34.69
**MDD021**	Moderate	39	Male	91.36932	10.01489	0.668231	54.84789	0.380653	0.599548	1.75728	49.85261	9.29594	0.56	55.69
**MDD022**	Mild	34	Male	84.18781	8.128752	0.694266	56.10535	0.400012	0.56249	1.551572	48.14266	8.974152	0.5	42.69
**MDD024**	Moderate	40	Male	63.81224	2.61	1.445153	67.02932	0.60168	0.329862	0.671964	41.83952	0.887803	0.325	46.69
**MDD025**	Moderate	27	Female	65.07617	3.156676	0.797899	62.97838	0.55697	0.459816	0.74578	44.1284	2.375607	0.335	69.69
**MDD026**	Moderate	27	Male	104.2635	23.111	0.550003	52.09763	0.198684	0.849268	3.03919	54.36505	13.55478	0.949333	86.69
**MDD027**	Mild	43	Female	68.01243	3.34	0.7488	63.42753	0.554611	0.489855	1.04151	44.70901	3.408658	0.358667	73.69
**MDD028**	Mild	30	Female	68.95181	4.43	0.736813	63.12753	0.545016	0.497792	1.054373	45.48193	3.477728	0.367	55.69
**MDD029**	Moderate	25	Female	104.044	22.38	0.470503	52.30119	0.202012	0.752279	2.866243	53.13307	13.55478	0.906667	47.69
**MDD030**	Moderate	25	Female	99.05418	18.06757	0.627133	52.92196	0.30626	0.676316	2.128763	50.9654	10.81274	0.673333	35.69

Optional Table 3: Previous Raw Data of 30 Subjects in MDD Group (Before Cut off participants of Age above 43 years old.)

SUBJECT CODE	TYPE	AGE	GENDER	HR	SDHR	MEAN NN	SDNN	HF	LF	LF/HF	MEAN PP	SDPP	CORTISOL	WCST
		(years)		(BPM)		(ms)	(ms)	(Hz)	(Hz)	(Hz)	(mmHg)	(mmHg)	(Ug/dL)	(%ctr)
**MDD001**	Moderate	34	Male	100.2094	22.082	0.600007	52.97838	0.285452	0.683723	2.61242	51.48396	11.56542	0.75	85.69
**MDD002**	Moderate	75	Female	93.95274	10.16018	0.653652	54.44789	0.367208	0.619397	1.846547	50.2726	9.797434	0.6	80.69
**MDD003**	Moderate	24	Female	94.00608	11.04298	0.640209	53.09763	0.365145	0.621951	1.898547	50.42157	9.885724	0.638667	84.69
**MDD004**	Moderate	34	Male	94.08978	12.03713	0.637004	53.92932	0.361839	0.642988	1.96417	50.65219	9.885724	0.64	47.69
**MDD005**	Moderate	44	Female	58.03927	2.05	1.716241	69.30119	0.65736	0.329131	0.454682	41.01607	0.651657	0.3	52.69
**MDD006**	Mild	59	Male	87.09429	7.110644	0.668406	55.92196	0.397263	0.569987	1.551707	48.5299	9.269897	0.549333	72.69
**MDD007**	Severe	33	Female	102.9251	21.10614	0.583077	52.65059	0.27251	0.744215	2.678893	52.26078	12.37064	0.853333	82.69
**MDD008**	Moderate	41	Female	89.683	8.393	0.680418	55.09763	0.393865	0.580973	1.595653	49.73191	9.29594	0.542667	85.69
**MDD009**	Moderate	41	Male	101.4409	21.10614	0.589044	52.6884	0.283049	0.701513	2.678893	52.12959	11.56542	0.8	51.69
**MDD010**	Moderate	60	Female	77.08861	4.6	0.722577	60.65059	0.48682	0.511135	1.11878	46.65278	4.106368	0.372	37.69
**MDD011**	Moderate	47	Female	80.13634	6.058379	0.702592	56.92932	0.478517	0.540803	1.33991	47.69736	5.557777	0.392	70.69
**MDD012**	Mild	54	Female	77.51854	5.054708	0.713502	58.6884	0.486055	0.52073	1.15174	46.72263	4.184179	0.377333	42.69
**MDD013**	Severe	40	Female	89.98518	9.9	0.6744	54.92196	0.389065	0.593317	1.75032	49.77614	9.797434	0.542667	98.69
**MDD014**	Moderate	22	Female	96.33837	16.40111	0.62906	53.97838	0.355791	0.67314	1.96417	50.96069	10.12024	0.672	36.69
**MDD015**	Moderate	35	Female	83.96518	7.009919	0.698949	56.10535	0.445193	0.549209	1.50184	47.82162	7.555456	0.439	58.69
**MDD016**	Mild	53	Female	64.01183	2.196754	1.002075	66.10535	0.575138	0.45768	0.458973	42.26418	2.112088	0.34	82.69
**MDD017**	Mild	42	Female	82.05428	6.08	0.701038	56.92932	0.47674	0.541071	1.37891	47.64733	5.818441	0.41	72.69
**MDD018**	Moderate	43	Female	64.95774	3.008218	1.336461	63.12753	0.565869	0.458841	0.49013	42.94451	1.903315	0.343	34.69
**MDD019**	Moderate	51	Female	83.05428	7.047828	0.699156	56.84789	0.416936	0.543161	1.44868	47.9655	7.934373	0.465	76.69
**MDD020**	Mild	48	Female	76.45083	4.102423	0.726826	61.22394	0.494119	0.504988	1.08138	46.36796	3.760119	0.367	88.69
**MDD021**	Moderate	39	Male	91.36932	10.01489	0.668231	54.84789	0.380653	0.599548	1.75728	49.85261	9.29594	0.56	55.69
**MDD022**	Mild	34	Male	84.18781	8.128752	0.694266	56.10535	0.400012	0.56249	1.551572	48.14266	8.974152	0.5	42.69
**MDD023**	Mild	45	Male	79.51354	5.241433	0.705183	58.6884	0.48427	0.52826	1.32503	46.8755	4.424176	0.379	73.69
**MDD024**	Moderate	40	Male	63.81224	2.61	1.445153	67.02932	0.60168	0.329862	0.671964	41.83952	0.887803	0.325	46.69
**MDD025**	Moderate	27	Female	65.07617	3.156676	0.797899	62.97838	0.55697	0.459816	0.74578	44.1284	2.375607	0.335	69.69
**MDD026**	Moderate	27	Male	104.2635	23.111	0.550003	52.09763	0.198684	0.849268	3.03919	54.36505	13.55478	0.949333	86.69
**MDD027**	Mild	43	Female	68.01243	3.34	0.7488	63.42753	0.554611	0.489855	1.04151	44.70901	3.408658	0.358667	73.69
**MDD028**	Mild	30	Female	68.95181	4.43	0.736813	63.12753	0.545016	0.497792	1.054373	45.48193	3.477728	0.367	55.69
**MDD029**	Moderate	25	Female	104.044	22.38	0.470503	52.30119	0.202012	0.752279	2.866243	53.13307	13.55478	0.906667	47.69
**MDD030**	Moderate	25	Female	99.05418	18.06757	0.627133	52.92196	0.30626	0.676316	2.128763	50.9654	10.81274	0.673333	35.69
